# *Saccharomyces cerevisiae* deficient in the early anaphase release of Cdc14 can traverse anaphase I without ribosomal DNA disjunction and successfully complete meiosis

**DOI:** 10.1242/bio.059853

**Published:** 2023-10-17

**Authors:** Christopher M. Yellman

**Affiliations:** Department of Biology, Georgetown University, 301 Regents Hall, 6411 Tondorf Road, Washington, DC 20007, USA

**Keywords:** Meiosis, Meiotic cell cycle, Meiotic chromosome segregation, Net1, Cdc14, rDNA disjunction, FEAR

## Abstract

Eukaryotic meiosis is a specialized cell cycle of two nuclear divisions that give rise to haploid gametes. The phosphatase Cdc14 is essential for meiosis in the yeast *Saccharomyces cerevisiae*. Cdc14 is sequestered in the nucleolus, a nuclear domain containing the ribosomal DNA, by its binding partner Net1, and released in two distinct waves, first in early anaphase I, then in anaphase II. Current models posit that the meiosis I release is required for ribosomal DNA disjunction, disassembly of the anaphase spindle, spindle pole re-duplication and counteraction of cyclin-dependent kinase, all of which are essential events. We examined Cdc14 release in *net1-6cdk* mutant cells, which lack six key Net1 CDK phosphorylation sites. Cdc14 release in early anaphase I was partially inhibited, and disjunction of the rDNA was fully inhibited. Failure to disjoin the rDNA is lethal in mitosis, and we expected the same to be true for meiosis I. However, the cells reliably completed both meiotic divisions to produce four viable spores. Therefore, segregation of the rDNA into all four meiotic products can be postponed until meiosis II without decreasing the fidelity of chromosome inheritance.

## Teaser

**Summary:** A yeast mutant completes both rounds of meiotic rDNA segregation during the Meiosis II phase.

## INTRODUCTION

In eukaryotes, the formation of gametes through meiosis requires the execution of a reductional chromosome segregation (meiosis I) and a subsequent equational division (meiosis II) (Ohkura, 2015). Diploid cells of the yeast *Saccharomyces cerevisiae* can undergo meiosis to produce an ascus containing four spores, each with the necessary haploid chromosome content. A variety of mutations studied in *S. cerevisiae*, including mutations of the *SLK19*, *SPO12* and *CDC14* genes, prevent cells from fully completing two meiotic chromosome divisions (Klapholz and Esposito, 1980b; Kamieniecki et al., 2000; Zeng and Saunders, 2000; Buonomo et al., 2003; Marston et al., 2003).

The Cdc14 protein undergoes a distinctive localization cycle during cell division, and the dynamics of its localization are important for regulating its activity. From G_1_ to metaphase, Cdc14 is stored within the nucleolus, attached to its binding partner Net1 in a chromatin silencing complex termed the REgulator of Nucleolar silencing and Telophase (RENT) (Shou et al., 1999; Straight et al., 1999; Visintin et al., 1999). In early anaphase of mitosis, Cdc14 is released from the RENT in a process that requires the Slk19 and Spo12 proteins and phosphorylation of Net1 by cyclin-dependent kinase (CDK) (Stegmeier et al., 2002; Azzam et al., 2004). Slk19 and Spo12 are also required for the release of Cdc14 during anaphase I of meiosis (Buonomo et al., 2003; Marston et al., 2003). The early anaphase release of Cdc14, Fourteen Early Anaphase Release (FEAR), is seen cytologically in both mitosis and meiosis I as a brief redistribution of the protein from the nucleolus throughout the nucleus without its export to the cytoplasm, followed by its return to the nucleolus near the end of anaphase (Yellman and Roeder, 2015). While non-essential for mitosis, FEAR is thought to be absolutely required for the completion of two rounds of meiotic division.

A separate, and essential pathway, the mitotic exit network (MEN), releases Cdc14 again at the end of anaphase of mitosis (Shou et al., 1999; Visintin et al., 1999). The MEN, in addition to releasing Cdc14, drives its export from the nucleus to the cytoplasm (Mohl et al., 2009). The MEN is also active in meiosis II, when Cdc14 is efficiently exported from the nucleus (Yellman and Roeder, 2015), but the pathway appears to be non-essential for meiosis (Kamieniecki et al., 2005; Attner and Amon, 2012; Renicke et al., 2017).

A variety of meiotic events are thought to depend on FEAR. These include segregation of the ribosomal DNA (rDNA) (Buonomo et al., 2003), disassembly of the anaphase I spindle (Marston et al., 2003; Kamieniecki et al., 2005), spindle pole re-duplication (Fox et al., 2017) and the counteraction of CDK to promote cell cycle exit and progression into the second round of meiotic division (Kamieniecki et al., 2005). The aforementioned events all share the property of being required for progression into meiosis II.

Due to specialized chromosome condensation requirements (Lavoie et al., 2003) and topological entanglements, segregation of the rDNA occurs relatively late in mitosis, after the cleavage of chromosomal cohesion proteins, and requires FEAR (D'Amours et al., 2004; Sullivan et al., 2004; Torres-Rosell et al., 2005). Failure to segregate the rDNA is lethal in mitosis, since the lagging chromosomal regions are severed by cytokinesis (Quevedo et al., 2012; Machín et al., 2016). rDNA segregation is also a late event in meiosis I, where it strictly requires the Slk19 and Spo12 proteins, likely due to their role in FEAR (Buonomo et al., 2003).

In yeast, all of the rDNA genes are encoded on the right arm chromosome XII as an array of ∼150 nearly identical transcriptional subunits, each ∼9.1 kilobases (kb) long (Petes, 1979; Kobayashi, 2014). In humans and mouse, the several hundred copies of the rDNA are distributed to loci on six different chromosomes, which nevertheless associate physically due to specialized chromatin regulation (Potapova and Gerton, 2019). Throughout eukaryotes, the chromatin of the rDNA is elaborately structured to balance the need for highly active transcription of the rDNA repeats by RNA polymerase I with the need to inhibit mitotic and meiotic recombination between repeats (Huang and Moazed, 2003; Li et al., 2014; Warmerdam and Wolthuis, 2019). The structure of the nucleolus lends it the properties of a phase separated body, and detection of nucleolar proteins reveals a region of the nucleus with distinct cytological boundaries (Brangwynne et al., 2011; Hult et al., 2017; Mangan et al., 2017; Lawrimore et al., 2021).

The Slk19 and Spo12 proteins have been studied extensively in both mitosis and meiosis, and a subset of their functions have been ascribed to their activation of FEAR. Mutation of the Net1 CDK sites also severely impairs mitotic FEAR, with surprisingly modest consequences (Azzam et al., 2004; Yellman and Roeder, 2015). While dispensable for mitosis, FEAR is thought to be important for progression through anaphase I of meiosis (Buonomo et al., 2003; Marston et al., 2003; Attner and Amon, 2012). We tested this hypothesis in order to clarify the critical meiotic events that depend upon FEAR.

A previous study using a *net1-6cdk-TEV-9MYC* allele showed that the CDK sites are important for the appropriate timing of meiosis I (Kerr et al., 2011). We found that C-terminal epitope fusions to Net1 inhibited cells from completing two meiotic divisions and forming tetrad asci, and therefore made an allele with no additional modifications. We characterized the *net1-6cdk* allele in meiosis and found that it impaired, but did not completely prevent FEAR. The CDK site mutations were, however, severely inhibitory to disjunction of the rDNA during meiosis I. To our surprise, the failure of rDNA disjunction had almost no impact on the ability of cells to traverse meiosis and produce four viable spores. We discuss the implications of these findings for our understanding of the role of Cdc14 and Net1 in meiotic rDNA disjunction and cell cycle progression.

## RESULTS

### The *net1-6cdk* mutations partially impair the release of Cdc14 during meiosis I

During anaphase of meiosis I, *S. cerevisiae* cells release Cdc14 into the nucleus from its storage location within the nucleolus. Since the *net1-6cdk* mutations severely inhibit FEAR in mitosis (Yellman and Roeder, 2015), we wanted to observe the phenotypes of these mutations in meiosis.

**Fig. 1.**
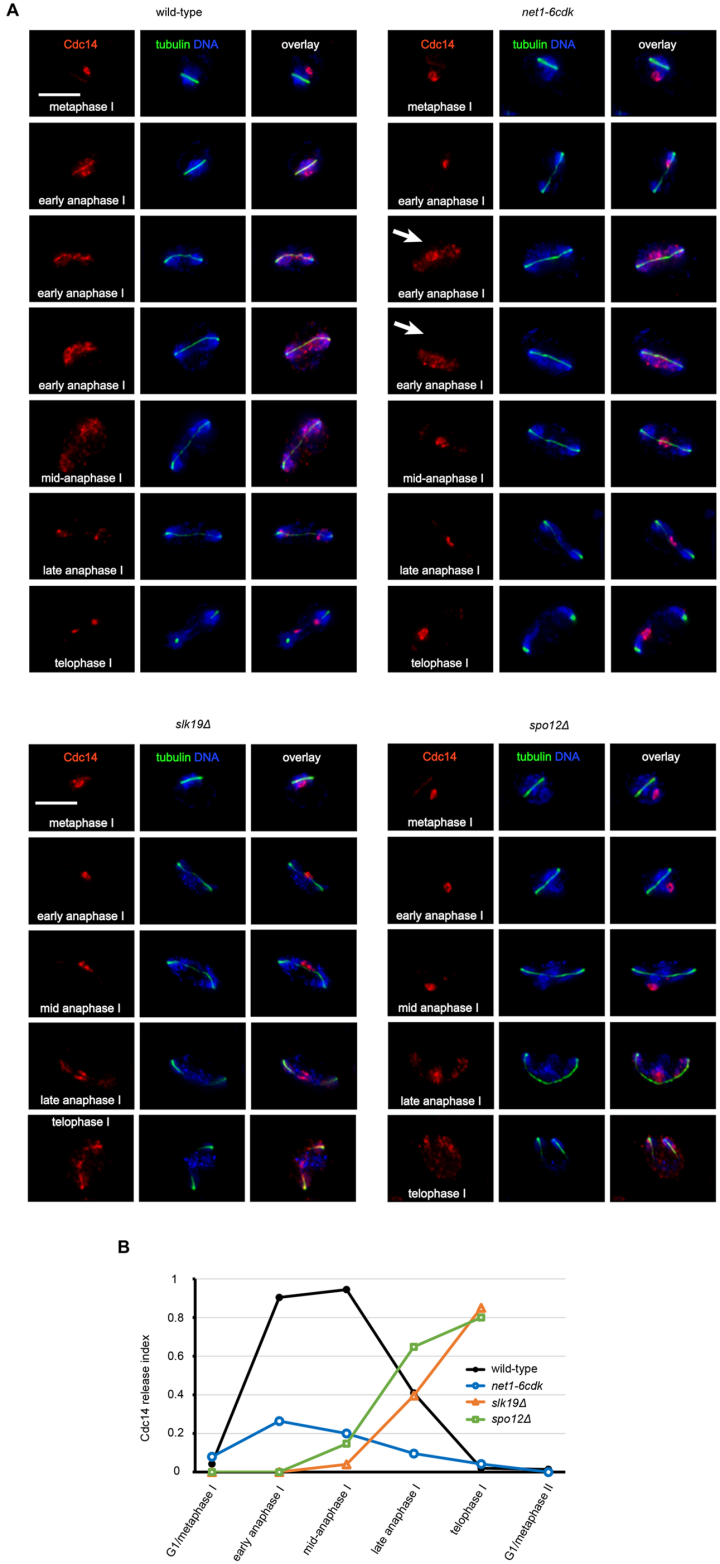
**The *net1-6cdk* mutations inhibit the meiosis I release of Cdc14 from the nucleolus.** Yeast cells were induced to enter meiosis and processed for indirect immunofluorescence microscopy of Cdc14-3Myc (red) and *α*-tubulin (green). DNA was stained with DAPI (blue). All images were acquired as z-series, deconvolved, and projected to a single plane. All data were gathered from images that had been processed and saved. Images were chosen to represent the distinct Cdc14 localization phenotypes. The genotypes and strains were wild-type (CMY 2508) *net1-6cdk* (CMY 2509), *slk19Δ* (CMY 2690) and *spo12Δ* (CMY 2691). (A) Release of Cdc14-3Myc from the nucleolus during meiosis I (scale bar: 5 μm). (B) Extent of Cdc14-3Myc release. An index of Cdc14-3Myc release was calculated by scoring cells that had undergone partial release as 0.5, and cells that had undergone full release as 1. Table S1 contains the numbers of cells in each category that comprise each data point in the graph.

Our wild-type cells behaved as expected, robustly releasing Cdc14 and reaching maximal release in early and mid-anaphase I. The images show examples of cells progressively releasing Cdc14 from early to mid-anaphase (Fig. 1A), and the graph summarizes the overall level of Cdc14 release (Fig. 1B). In contrast, less than half of *net1-6cdk* cells observed in anaphase I detectably released Cdc14, and only one sixth released Cdc14 as robustly as wild-type. Although it is atypical for *net1-6cdk* cells to robustly release Cdc14, we included examples of them with Cdc14 in a state of partial or near complete release (indicated in the images with white arrows) to illustrate the range of phenotypes. The release was often partial, with a substantial amount of the protein remaining in a concentrated region. By late anaphase I, both wild-type and *net1-6cdk* cells had begun to relocalize Cdc14 efficiently to the nucleolus. The primary data derived from scoring individual images, and the numbers of cells analyzed for each data point, are in Table S1.

For comparison, we analyzed mutants lacking the Slk19 and Spo12 proteins, both of which have been extensively studied and are known to be required for the release of Cdc14. Both *slk19Δ* and *spo12Δ* mutations severely inhibited the release of Cdc14 in early anaphase (Fig. 1). Unlike wild-type and *net1-6cdk* cells, however, cells of both deletion genotypes proceeded to release some Cdc14 during late anaphase, and many cells reached telophase with Cdc14 partially released (Fig. 1 and Table S1). To our knowledge, this delayed release has not previously been reported, and would be difficult to see except in the type of intensive single-cell imaging we carried out. The meiotic phenotypes of *slk19Δ* and *spo12Δ* cells are more complex and extensive than what we have observed for *net1-6cdk*, including the execution of chromosome segregation patterns that are a mix of MI and MII (Kamieniecki et al., 2000; Zeng and Saunders, 2000). The late meiosis I release of Cdc14 we saw in *slk19Δ* and *spo12Δ* cells is similar to what previous reports have described as partial meiosis II chromosome segregation on a meiosis I spindle.

In summary, the *net1-6cdk* mutations impaired the anaphase I release of Cdc14, although less severely than *slk19Δ* and *spo12Δ*. Besides the six phosphorylation sites we mutated, Net1 has additional CDK sites which are not critical for Cdc14 release, but may contribute modestly to the release of Cdc14 (Azzam et al., 2004). We do not know how much released Cdc14 might be required for its meiosis I activities. Therefore, we must consider that the hypomorphic *net1-6cdk* allele may compromise different meiosis I activities of Cdc14 to different degrees.

### The *net1-6cdk* mutations severely inhibit disjunction of the rDNA during meiosis I

Disjunction of the rDNA into two distinct masses normally takes place in late anaphase I as spindle forces segregate the chromosomes. Mitotic studies in both yeast and human cells have shown that Cdc14 is required for silencing transcription within the rDNA (Clemente-Blanco et al., 2009), and for loading condensin proteins into the rDNA to assist in resolution of interchromosomal linkages and enable chromosome segregation (D'Amours et al., 2004; D'Ambrosio et al., 2008; Daniloski et al., 2019).

**Fig. 2.**
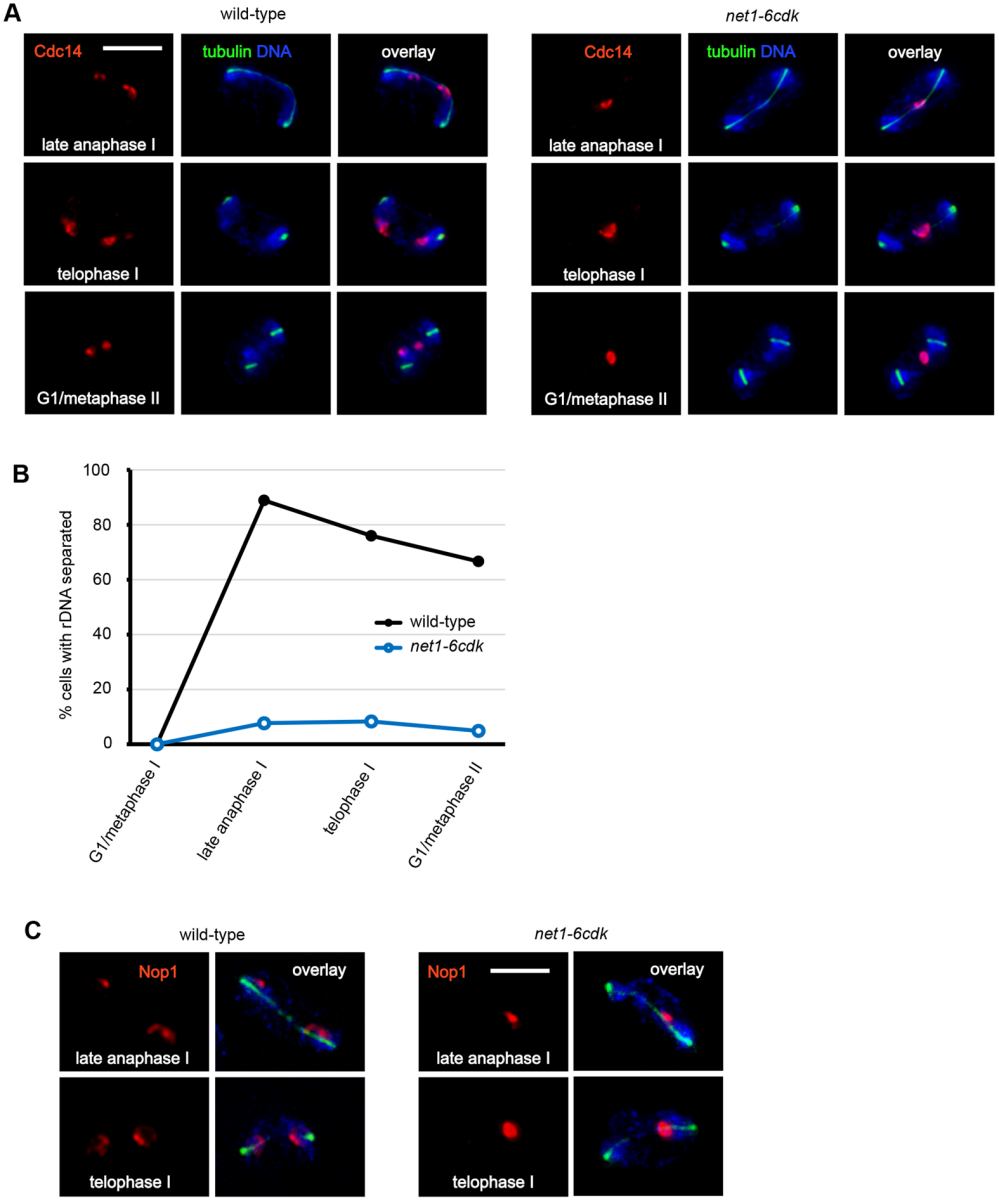
**The *net1-6cdk* mutations inhibit segregation of the rDNA in meiosis I.** Wild-type (CMY 2508) and *net1-6cdk* (CMY 2509) cells were induced to enter meiosis and processed for indirect immunofluorescence microscopy as described for Fig. 1. All data were collected from saved images. Scale bar: 5 μm. (A) Representative images of rDNA separation as observed by Cdc14-3Myc staining from late meiosis I through early meiosis II. (B) Quantification of Cdc14-3Myc separation into one or two masses. (C) Representative images of Nop1 localization during late anaphase and early telophase of meiosis I. Table S2 contains the numbers of cells in each rDNA separation category that comprise each data point in the graph.

Since *net1-6cdk* cells had efficiently returned Cdc14 to the nucleolus by late anaphase I, we were able to infer the position of the nucleolus from Cdc14 localization. In striking contrast to wild-type, the majority of *net1-6cdk* cells failed to disjoin the nucleolus (Fig. 2A and B). During the late stages of spindle elongation and into telophase, when spindle breakdown was under way, Cdc14 remained in a single mass positioned between the divided chromosomal DNA.

Because *slk19Δ* and *spo12Δ* cells had mostly released Cdc14 during anaphase spindle elongation, we were unable to observe the positions of their nucleoli, but previous work has shown that they fail to disjoin the rDNA (Buonomo et al., 2003). By meiotic metaphase II, the vast majority of *net1-6cdk* cells still maintained the nucleolus in a single mass. The quantitative data derived from scoring individual images are in Table S2. There was some re-grouping of the nucleoli into a single mass in metaphase II wild-type cells, probably due to the relaxation of spindle tension.

For additional confirmation of rDNA positioning, we examined the localization of the nucleolar protein Nop1 (Fig. 2C). All of the wild-type or *net1-6cdk* cells that we observed up to mid-anaphase I had a single mass of Nop1 as expected, since nucleolar segregation occurs in late anaphase I. When we looked at cells in late anaphase or telophase I, however, all seven wild-type cells that we observed had separated Nop1 into two masses, while none of the seven *net1-6cdk* cells had done so. In summary, localizations of Nop1 and Cdc14 were similar, and the vast majority of *net1-6cdk* cells traversed meiosis I without disjoining their rDNA. An important caveat to this conclusion is that, without a second nucleolar marker, we cannot exclude the possibility that some Cdc14 was outside of the nucleolus. For this reason, we may have underestimated the extent of Cdc14 release.

### Meiosis I spindles appear normal in *net1-6cdk* cells

Cdc14 is required for microtubule spindle growth and stability in meiosis, and both Slk19 and Spo12 are required for normal meiotic spindle morphology (Buonomo et al., 2003; Havens et al., 2010). Slk19 is required for spindle midzone stability independent of its role in Cdc14 release (Havens et al., 2010), and in both *slk19Δ* and *spo12Δ* mutants, the anaphase I spindle persists when progression into meiosis II requires that it be disassembled (Buonomo et al., 2003).

**Fig. 3.**
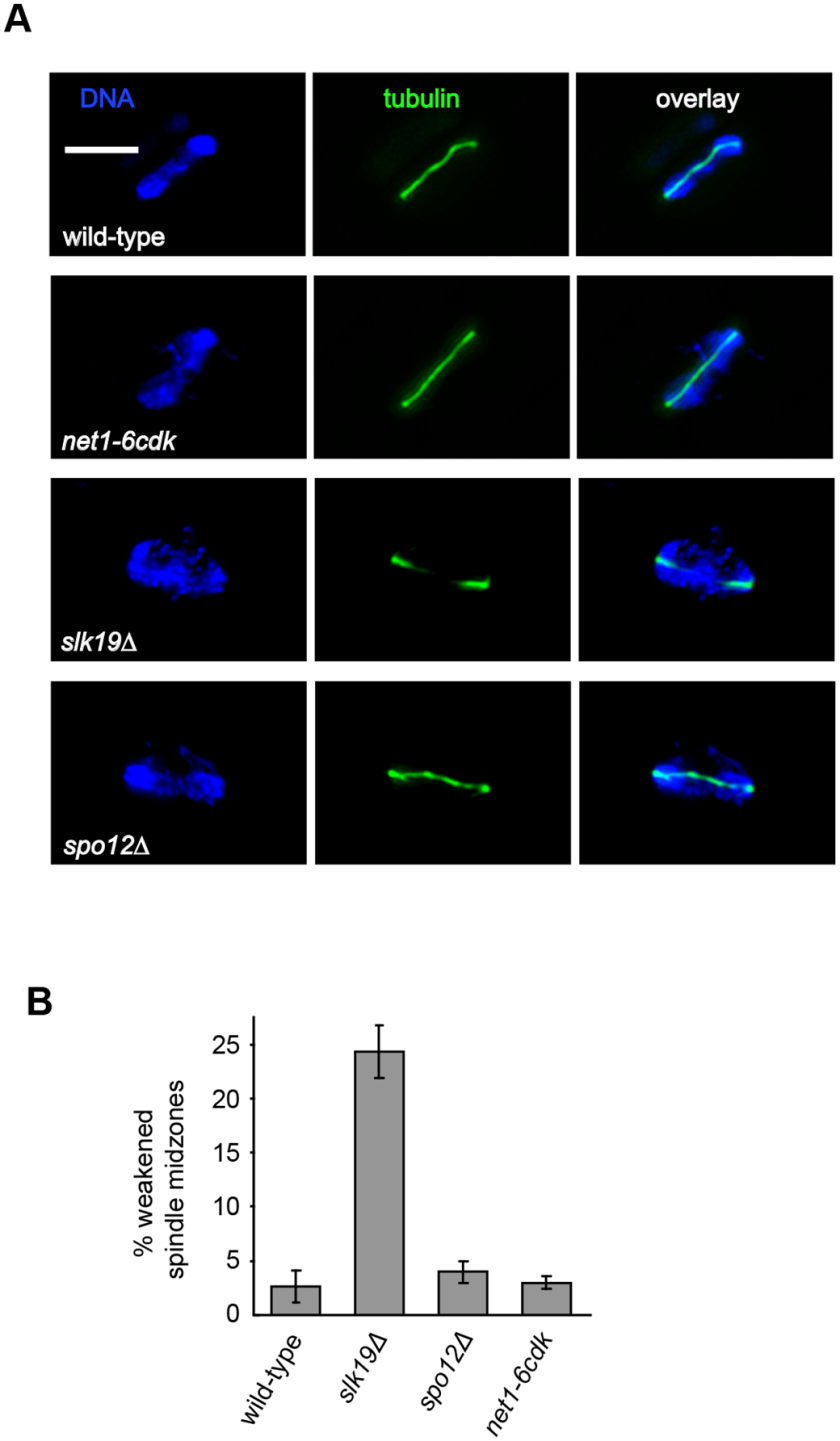
***net1-6cdk* mutants have no apparent defects in the meiosis I spindle midzone.** Wild-type (CMY 1901), *net1-6cdk* (CMY 1903), *slk19Δ* (CMY 2347) and *spo12Δ* (CMY 2348) cells were induced to enter meiosis and processed for indirect immunofluorescence microscopy as described for Figs S1 and S2. (A) Representative images of mid-anaphase I spindles (scale bar: 5 μm). (B) Quantification of the weakened spindle midzone phenotype characteristic of *slk19* cells. The experiment was performed in triplicate, scoring a total 300 cells of each genotype. Error bars represent the standard error of the mean.

We considered that the failure of *net1-6cdk* cells to disjoin the rDNA could result from impairment of the spindle. In our observations of Cdc14 localization, the anaphase I spindles of *net1-6cdk* cells had appeared normal, with spindle disassembly proceeding as the cells approached telophase (Figs 1A and 2A). As previously reported, *slk19Δ* cells often had a weakened spindle midzone and short spindles, a phenotype that was particularly evident in mid-anaphase I. We confirmed that this phenotype was unique to the *slk19Δ* cells, and that the spindle midzones of *net-6cdk* and *spo12Δ* cells were similar to wild-type (Fig. 3). Our analysis did not provide any insight into spindle dynamics in the mutants, since the data were snapshots from asynchronous populations. In summary, *net1-6cdk* cells have an apparently normal meiosis I spindle which should supply the force necessary to disjoin the rDNA, and is disassembled as cells complete meiosis I.

### Nuclear division, spore formation and spore viability are normal in *net1-6cdk* cells

The release of Cdc14 from the nucleolus and disjunction of the rDNA are both thought to be required for the accurate completion of two rounds of meiotic chromosome segregation and the production of four viable spores. Deficiencies in the release of Cdc14 from the nucleolus by *slk19Δ* and *spo12Δ* mutants have been associated with failure to complete meiosis I (Kamieniecki et al., 2000; Zeng and Saunders, 2000; Buonomo et al., 2003; Marston et al., 2003). The *slk19Δ* mutant undergoes mixed reductional and equational chromosome segregation, with defects in both spindle midzone integrity and centromeric sister chromatid cohesion (Kamieniecki et al., 2000; Havens et al., 2010), while *spo12* mutants primarily complete a single equational division resembling meiosis II (Klapholz and Esposito, 1980a,b).

**Fig. 4.**
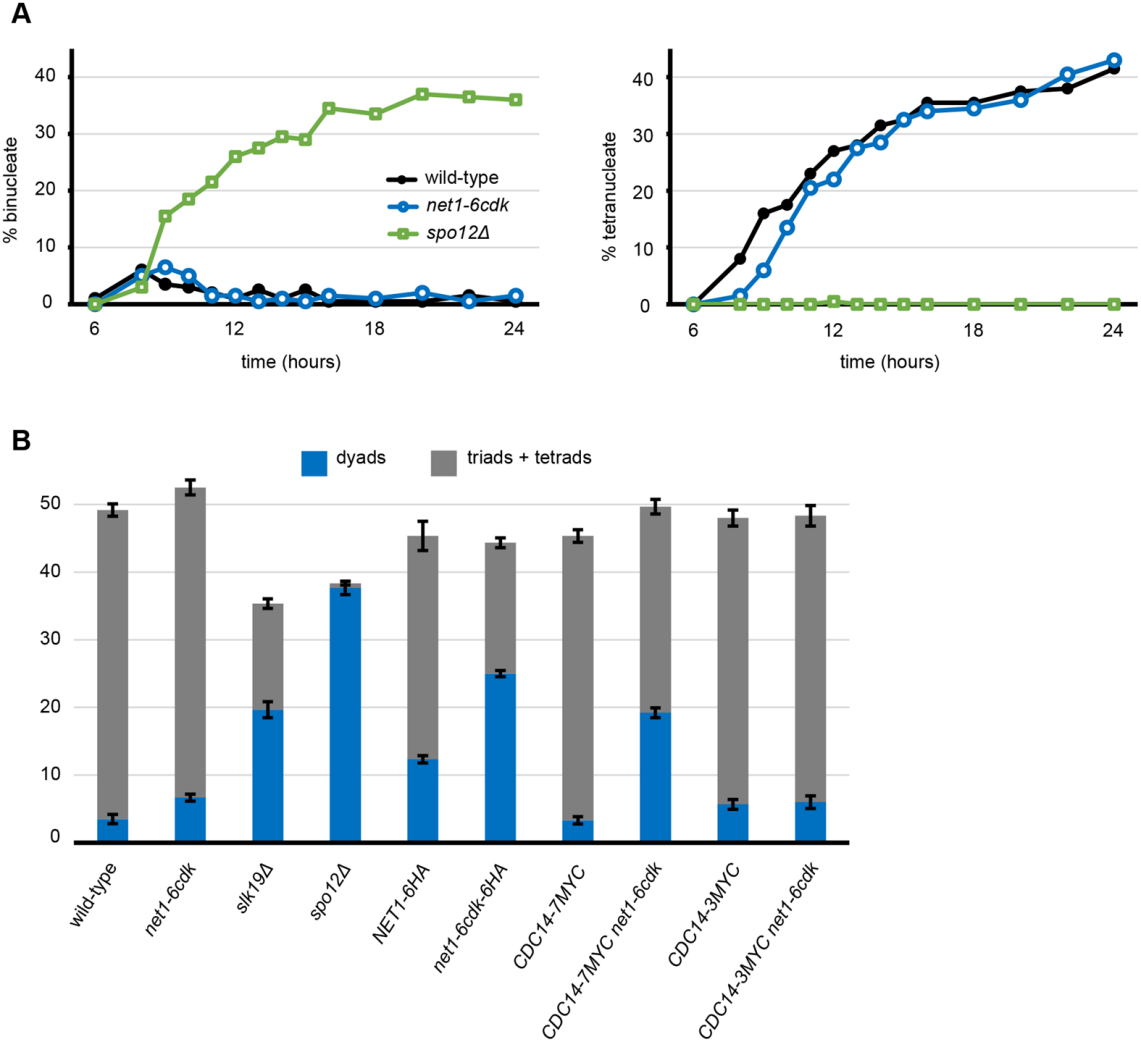
**Cells with the *net1-6cdk* mutations complete two meiotic nuclear divisions and form spores.** Cells were stained with DAPI to observe the DNA. Nuclear division was scored as cells completed meiosis I to form binucleates, then meiosis II to form tetranucleates. The genotypes and strains were wild-type (CMY 1901), *net1-6cdk* (CMY 1903), *slk19Δ* (CMY 2347), *spo12Δ* (CMY 2348), *NET1-6HA* (CMY 1992), *net1-6cdk-6HA* (CMY 2086), *CDC14-7MYC* (CMY 2150), *CDC14-7MYC net1-6cdk* (CMY 2151), *CDC14-3MYC* (CMY 2508) and *CDC14-3MYC net1-6cdk* (CMY 2509). (A) Nuclear division as observed by DAPI staining. Data are the average of two replicates, and a total of 200 cells were counted for each data point. (B) Spore formation after 32 h, as observed by phase contrast microscopy, scoring at least 300 cells of each genotype. Error bars represent the standard error of the mean.

We explored the ability of the *net1-6cdk* mutant to complete both meiotic chromosome divisions, analyzing three indicators of successful meiosis: nuclear division, spore formation and spore viability. We observed progression through the two nuclear divisions of meiosis by counting the formation of binucleate and tetranucleate cells during a 24-h time course (Fig. 4A). *net1-6cdk* cells initiated meiotic nuclear division with only a slight delay relative to wild-type, ultimately forming similar levels of tetranucleates. Consistent with previous observations, *slk19Δ* cells formed a mixture of binucleate and trinucleate cells and *spo12Δ* almost exclusively formed binucleates (Buonomo et al., 2003), albeit with kinetics similar to wild-type tetranucleate formation (Fig. 4A). Since the *slk19Δ* phenotype is difficult to assess and has previously been described, we did not include it in the analysis.

After 36 h in sporulation medium *net1-6cdk* cells had formed tetrad asci with a similar efficiency to wild-type cells, although the mutant formed slightly more dyad asci than the wild-type (Fig. 4B). It is hard to definitively distinguish triads from tetrads, but we informally observed that the spores formed by *slk19Δ* were often of unequal sizes and appeared to contain a high proportion of dyads and triads. As expected, *spo12Δ* cells almost exclusively formed dyads.

We examined spore viability by dissecting asci, and the data are reported in Table 1. The viability of spores from homozygous *net1-6cdk* cells, and a heterozygous mutant strain that we also dissected, were as high as wild-type cells at 97%. We also analyzed the viability of spores from *slk19Δ* and *spo12Δ* dyad asci. The *slk19Δ* mutant produced 44% viable spores, while *spo12Δ* spore viability was closer to normal at 86%. The simplest explanation for low spore viability is inaccurate chromosome segregation and conversely, the formation of viable spores by the *net1-6cdk* mutant indicated that all chromosomes were faithfully segregated.

**Table 1.**
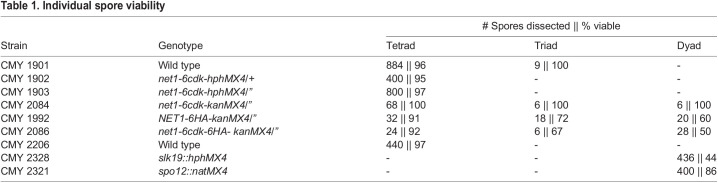
Individual spore viability

**Fig. 5.**
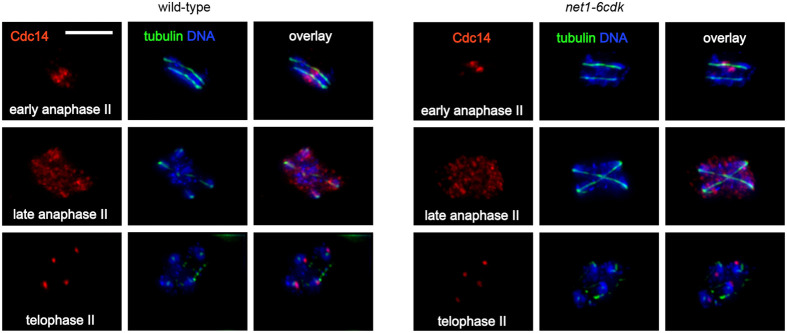
**The meiosis II release of Cdc14 is normal in *net1-6cdk* cells.** Representative images of cells in meiosis II (scale bar: 5 μm). Wild-type (CMY 2508) and *net1-6cdk* (CMY 2509) cells were induced to enter meiosis and processed for indirect immunofluorescence microscopy as described for Figs 1 and 2.

Formation of the expected number of viable spores requires completion of meiosis II. Our images of cells in meiosis II revealed a robust late anaphase release of Cdc14 in both wild-type and *net1-6cdk* cells (Fig. 5). In summary, the *net1-6cdk* mutant produced only slightly elevated levels of dyad asci, and otherwise was highly proficient in the completion of meiosis.

### C-terminal epitope fusions to Cdc14 and Net1 compromise the activity of the proteins

During our investigation of dyad formation, we observed that a C-terminal Net1-6HA epitope fusion caused elevated levels of dyads to form, an effect that was synergistic with the *net1-6cdk* mutations, leading to even higher levels of dyads (Fig. 4B). Recent work has revealed a role for the C-terminus of Net1 in activating RNA polymerase I transcription (Hannig et al., 2019). The C-terminal Cdc14-7MYC epitope fusion to Cdc14 was also synergistic with *net1-6cdk* for dyad formation, while Cdc14-3MYC had only a very weak effect. It has been reported that a C-terminal Cdc14-3HA epitope fusion strongly inhibits meiosis in yeast cells of the SK-1 background (Alonso-Ramos et al., 2020 preprint). The effects we observed were modest, indicating that the penetrance of such phenotypes depends strongly on the genetic background. Nevertheless, it appears that the C-termini of both Cdc14 and Net1 have activities that affect meiotic chromosome segregation. The study of C-terminal mutations of these proteins may yield additional insight into these sensitive functions.

## DISCUSSION

The early anaphase release of Cdc14 has been linked to a variety of events necessary for the termination of mitosis (Machín et al., 2016; Manzano-López and Monje-Casas, 2020) and meiosis I. However, it has been difficult to ascertain whether FEAR is directly responsible for the coincident events of chromosome segregation, spindle disassembly, spindle pole reduplication and CDK downregulation, particularly since the MEN becomes active soon after anaphase and is functionally redundant for the completion of those events. The use of highly specific mutations is one approach to untangling this problem, and we have previously used the *net1-6cdk* allele to show that FEAR does not have a significant role in mitotic nuclear division, spindle morphogenesis and mitotic exit – events closely associated with cell cycle progression (Yellman and Roeder, 2015). With the caveat that *net1-6cdk* is hypomorphic for meiotic FEAR, our current findings open the possibility that, in meiosis, bulk nuclear division, spindle morphogenesis, and progression into meiosis II are independent of FEAR. Alternatively, cells may be sensitive to the amount of Cdc14 that is released, and *net1-6cdk* may not limit the release sufficiently to compromise all Cdc14 functions. There are many additional CDK candidate phosphorylation sites in Net1 (Azzam et al., 2004), and combinations of mutations that we did not analyze might yield alleles that severely inhibit meiotic FEAR.

We found only one strong phenotype for the *net1-6cdk* allele in meiosis, a phenotype it shares with the classic FEAR mutants *slk19Δ* and *spo12Δ* – failure to disjoin the rDNA in meiosis I. From studies of mitotic cells we know that rDNA disjunction depends on two important activities of Cdc14 related to rDNA chromatin organization: condensin loading (Freeman et al., 2000; D'Amours et al., 2004; Sullivan et al., 2004; Wang et al., 2004) and control of transcription within the rDNA (Clemente-Blanco et al., 2009; 2011). Our current findings suggest that the phospho-regulation of Net1, while it is important for the retention and release of Cdc14 from the RENT complex, primarily affects rDNA chromatin organization rather than cell cycle progression *per se*. The *slk19Δ* and *spo12Δ* alleles delay the phosphorylation of Net1, at least at the T212 CDK site (Azzam et al., 2004). Therefore, it is an open possibility that Slk19 and Spo12 similarly promote rDNA disjunction by stimulating phosphorylation of Net1.

Disjunction of the rDNA occurs in meiosis I and was thought to be important for faithful chromosome segregation. In mitosis, failure to disjoin the rDNA is lethal, so how can it be non-lethal in meiosis I, as we found it to be in *net1-6cdk* mutant cells? In yeast, both meiotic nuclear divisions occur within the mother cell cytoplasm, without the accompanying division of the nuclear membrane or cytokinesis that occurs in mitosis (Moens and Rapport, 1971; Maier et al., 2007). In metazoans, the nuclear envelope breaks down during meiosis (Varberg and Jaspersen, 2018). Therefore, until gametes are packaged at the end of meiosis, there is no physical structure to sever the lagging chromosomal domains.

Cells with *net1-6cdk*, *slk19Δ* or *spo12Δ* mutations fail to disjoin the rDNA during meiosis I, but they must eventually do so to form viable spores, and even the deletion mutants form some viable spores. *net1-6cdk* cells proceed efficiently into meiosis II, and in late anaphase of meiosis II they release Cdc14 similarly to wild-type. If Cdc14 release is the critical event that drives rDNA disjunction, then its release in meiosis II seems to be fully redundant and able to disjoin the rDNA loci of both bivalent homologous chromosomes (meiosis I disjunction) and sister chromatids (meiosis II disjunction). Likewise, the late partial release of Cdc14 in *slk19Δ* and *spo12Δ* cells is likely to be MEN-dependent and stimulatory to rDNA disjunction, consistent with the ability of those mutants to form some viable haploid products (Table 1).

Our overall conclusions about the release of Cdc14 in early anaphase of meiosis I are similar to what we previously found for mitosis (Yellman and Roeder, 2015). Cdc14 release may be dispensable except for its role in rDNA disjunction, and inhibition of the release causes a non-lethal delay in chromosome segregation. Cell cycle regulation by Cdc14 has a long history of investigation (Hartwell and Smith, 1985; Visintin et al., 1998; Bloom et al., 2011), but the general model for Cdc14 in counteraction of CDK activity has recently been called into question (Powers and Hall, 2017). We hope our findings will help distinguish the roles of Cdc14 in the cell cycle from other critical but indirectly related activities.

## MATERIALS AND METHODS

### Yeast strains

All strains of *S. cerevisiae* used in this study were of the W303 background. Strain names and their genotypes are listed in Table 2. Mutations and epitope fusion alleles that were not part of the background genotype are described in detail and listed in Table 3. The *net1-6cdk* mutation has previously been fully sequenced (Yellman and Roeder, 2015). All epitope fusions were C-terminal and have also been previously sequenced across the region containing the C-termini of the genes and the multiple epitope arrays (same reference).

**Table 2.**
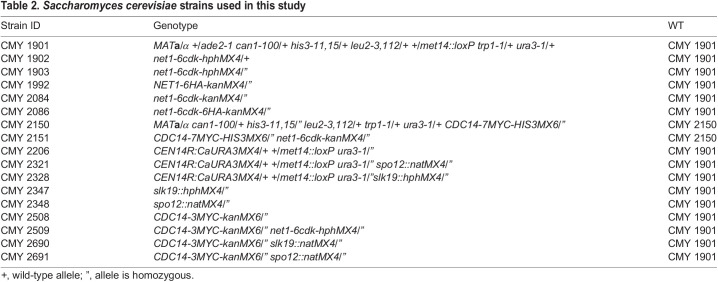
*Saccharomyces cerevisiae* strains used in this study

**Table 3.**
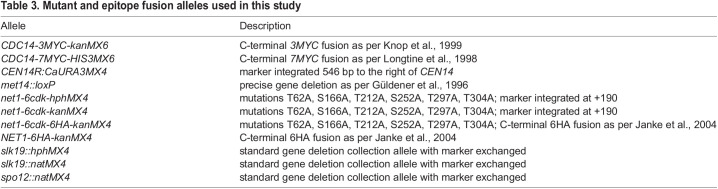
Mutant and epitope fusion alleles used in this study

### Media and growth conditions

YPAD was used for pre-sporulation growth of *S. cerevisiae* cultures, and SC dropout media were used to score the genetic markers of dissected tetrads. YPAD, SC dropouts and liquid sporulation media were standard and have been previously described (Amberg et al., 2005). A temperature of 30°C was used for both routine growth and induction of meiosis.

### Induction of meiosis

To induce entry into meiosis, cells were grown in YPAD medium at 30°C to early stationary phase (1–2×10^8^ cells/ml), washed once with water, diluted to an OD_600_ of 1.0 in fresh sporulation medium and incubated at 30°C on a platform shaker. The time of transfer to sporulation medium was considered time zero, and cells used for imaging were sampled 10–12 h later.

### Whole cell mounts for microscopy

Cells were fixed in the growth medium with 3.7% formaldehyde at room temperature for 30 min and their cell walls digested with a solution containing 50 μg/ml zymolyase and 1:50 glusulase (Perkin Elmer). The digested cells were mounted on poly-L lysine coated slides to immobilize them for microscopy. Slide preparation for all samples was finished by adding mounting medium containing DAPI to stain the DNA.

### Observation of nuclear division

To observe nuclear division, cells were fixed for 1 h in the growth medium with 3.7% formaldehyde at room temperature, and for an additional hour with 50% ethanol before samples were mounted with DAPI.

### Indirect immunofluorescence for protein detection and DNA

All localizations of Cdc14 were performed by indirect immunofluorescence of a Cdc14-3Myc fusion protein. Staining was performed with the mouse 9E10 monoclonal anti-Myc antibody (Covance) at 1:600 in PBS/1% BSA for 3 h at room temperature, followed by anti-mouse CY3 (Jackson ImmunoResearch) secondary antibody at 1:600 for 1 h at room temperature. α-tubulin was stained with the rat YOL 1/34 monoclonal antibody, followed by anti-rat FITC (Jackson ImmunoResearch). The native Nop1 protein was stained with the MCA-28F2 mouse monoclonal antibody (EnCor Biotechnology), followed by anti-mouse CY3 (Jackson ImmunoResearch).

### Microscopy and image processing

Images were taken using an Olympus UPlanS APO 100X objective lens (numerical aperture 1.40) on an Olympus IX-70 microscope equipped with the DeltaVision RT (Applied Precision, GE Healthcare) imaging system. Z-section image series were collected at 0.2 μM intervals over a total of 3–4 μM through the center of the cells. All protein and DNA localization was done with images that had been deconvolved using SoftWoRx (Applied Precision, GE Healthcare).

The images were converted into RGB.tif files using the image processing software Fiji. To reduce background, the red, green and blue channel levels were all set to a minimum level of 25 on a scale from 1 to 255 using Photoshop.

### Determination of cell cycle stage

The intranuclear portion of the spindle was used to determine the cell cycle stages of individual cells. Cells undergoing nuclear division were divided into the following categories: metaphase (short, thick spindle with round DNA mass), early anaphase (spindle and DNA mass slightly elongated), mid-anaphase (intermediate length spindle and elongated DNA mass), late anaphase (fully elongated spindle with a weakened mid-zone and the majority of DNA in two separate masses), and telophase (spindle undergoing disassembly with the DNA in two distinct masses). Meiosis I and II were distinguished by the presence of one or two spindles, respectively.

### Quantification of Cdc14 localization

Cdc14 was determined to be nucleolar when it was tightly localized to a part of the nucleus with an attenuated DAPI signal (characteristic of the rDNA) and nuclear when its distribution broadened to include the strongly DAPI-stained area. All protein localization data were gathered from saved images of individual cells that had been processed identically.

### Dissection of meiotic asci for tetrad analysis

Tetrads were dissected according to standard methods (Amberg et al., 2006).

### Statistical analysis

For Figs 1 and 2, the numbers of cells sampled for each data point of the graphs is summarized in Tables S1 and S2. The raw images for Figs 1 and 2 were collected in several batches, but not formally tracked as replicates. Numbers of cells sampled for all other figures are included in the figure legends themselves, and the experiments performed in at least three replicates. Control and experimental samples of all genotypes were processed in parallel to minimize the chances of batch effects. Mean values were calculated and standard error of the means applied as a statistical test to generate error bars.

## Supplementary Material

10.1242/biolopen.059853_sup1 Supplementary information

Table S1. Cdc14-3Myc release from the nucleolus

Table S2. rDNA disjunction by Cdc14-3Myc localization
