# Supplementary material for: Saccharomyces cerevisiae deficient in the early anaphase release of Cdc14 can traverse anaphase I without ribosomal DNA disjunction and successfully complete meiosis
Source: Biol Open. 2023 Oct 17;12(10):bio059853. doi: 10.1242/bio.059853 (PMC10621906; doi:10.1242/bio.059853)
Supplement: Supplementary information [file biolopen-12-059853-s1.pdf]

Table S1. Cdc14-3Myc release from the nucleolus

Click here to download Table S1

Table S2. rDNA disjunction by Cdc14-3Myc localization

Click here to download Table S2

Biology Open • Supplementary information
